# Quantitative Assessment of Optimal Bone Marrow Site for the Isolation of Porcine Mesenchymal Stem Cells

**DOI:** 10.1155/2017/1836960

**Published:** 2017-04-30

**Authors:** J. S. McDaniel, B. Antebi, M. Pilia, B. J. Hurtgen, S. Belenkiy, C. Necsoiu, L. C. Cancio, C. R. Rathbone, A. I. Batchinsky

**Affiliations:** ^1^United States Army Institute of Surgical Research, Fort Sam, Houston, TX, USA; ^2^The Geneva Foundation, Tacoma, WA, USA

## Abstract

*Background*. One of the most plentiful sources for MSCs is the bone marrow; however, it is unknown whether MSC yield differs among different bone marrow sites. In this study, we quantified cellular yield and evaluated resident MSC population from five bone marrow sites in the porcine model. In addition, we assessed the feasibility of a commercially available platelet concentrator (Magellan® MAR01™ Arteriocyte Medical Systems, Hopkinton, MA) as a bedside stem cell concentration device. *Methods*. Analyses of bone marrow aspirate (BMA) and concentrated bone marrow aspirate (cBMA) included bone marrow volume, platelet and nucleated cell yield, colony-forming unit fibroblast (CFU-F) number, flow cytometry, and assessment of differentiation potential. *Results*. Following processing, the concentration of platelets and nucleated cells significantly increased but was not significantly different between sites. The iliac crest had significantly less bone marrow volume; however, it yielded significantly more CFUs compared to the other bone marrow sites. Culture-expanded cells from all tested sites expressed high levels of MSC surface markers and demonstrated adipogenic and osteogenic differentiation potential. *Conclusions.* All anatomical bone marrow sites contained MSCs, but the iliac crest was the most abundant source of MSCs. Additionally, the Magellan can function effectively as a bedside stem cell concentrator.

## 1. Background

Bone marrow contains a heterogeneous mixture of cells, which provides the basis for its robust regenerative capacity. Mesenchymal stem cells (MSCs) represent a small population of plastic-adherent bone marrow stem cells that at a minimum possess the ability to differentiate along the osteogenic, chondrogenic, and adipogenic lineages [1]. Aside from their direct and proposed transdifferentiation capacity [2], MSCs have been shown to possess antiapoptotic, antibacterial, and anti-inflammatory capabilities, predominately elicited via bioactive mediators which they produce [3, 4]. Due to their versatile regenerative properties, MSCs have been at the forefront of cell-based clinical trials during the last decade [5]. Utilization of these cells in chronic conditions is most often translated from preliminary work in small animal models [6]. We envision an increase in stem cell use in trauma and critical care and believe that large animal models can address many of the potential problems associated with stem cell therapy. Animal studies that more closely recapitulate the physiological sequelae following injury facilitate the extrapolation of their data to humans.

Clinical administration of MSCs requires a large quantity of cells (1–10 million cells per kilogram) [5, 7]. Since MSCs are present in extremely low concentrations in the bone marrow (~0.001%–0.01%) [8], it is imperative to maximize their recovery from the subject for allogeneic and autologous treatments. Traditionally, isolation of MSCs is achieved through the initial separation of the mononuclear cell (MNC) population using density-gradient centrifugation (e.g., Ficoll-Paque) of the whole bone marrow aspirate. The MNCs are subsequently cultured at low densities, and within 2-3 weeks, the plastic-adherent cells form colonies, termed “colony-forming unit fibroblasts” (CFU-F), as each colony is thought to originate from a single MSC [9, 10]. This process is relatively lengthy and has been known to recover only 15–30% of the initial cell population [11–14]. Other processing techniques have been reported to be more efficient in the recovery of MSCs, such as direct plating of the bone marrow [2, 15, 16] and red-blood-cell removal prior to plating [17, 18]. Although these approaches have the advantage of capturing a larger set of the bone marrow cell population, without losing MSCs to subsequent postprocessing steps, they are still laborious and do not preserve the naïve bone marrow milieu that may be regenerative in nature (e.g., growth factors and cytokines).

Another method to isolate MSCs is through a bedside cell concentration device that yields a bone marrow concentrate (BMC) specimen [19–26]. One advantage of such a device is the quick centrifugation step used to generate the BMC, which bypasses the time-consuming step of cell culture and expansion and enables immediate administration to the patient that may translate to clinical significance. Depending on the particular device, the BMC may contain different cell populations, other than MSCs, and a variety of growth factors that may aid in the overall regenerative process [27–30]. For this purpose, we aimed to evaluate stem cell isolation using a point-of-care device (Magellan MAR01 System, Hopkinton, MA).

Since bone marrow MSCs reside in the trabecular component of flat and long bones, they can be harvested from multiple anatomical locations. In humans, bone marrow MSCs are typically isolated from the iliac crest, while in porcine, various anatomical sites (sternum, proximal tibia, femur, and iliac crest) have been used without systematic justification [31–35]. In this study, we characterized the relationship between donor sites and cell yield in a porcine model with the aim of autologous administration of MSCs. We sought to characterize cells from all locations, and based on existing literature in humans, it hypothesized that the iliac crest provides the most abundant source of cells for translational use. In addition, we assessed the feasibility of bedside stem cell concentration using the Magellan (Arteriocyte Medical Systems, Hopkinton, MA). This platelet concentration device is capable of concentrating white blood cells within 30 minutes after sampling the bone marrow. For this reason, we assessed the efficiency of the Magellan as a potential stem cell concentrator for critical-care scenarios requiring autologous cell-based interventions.

## 2. Materials and Methods

This study was approved by the US Army Institute of Surgical Research (an AAALAC-accredited facility) Animal Care and Use Committee. It was conducted in compliance with the Animal Welfare Act and the implementing Animal Welfare Regulations and in accordance with the principles of the Guide for the Care and Use of Laboratory Animals.

### 2.1. Surgical Procedure

Five Yorkshire female cross-bred pigs (42.5 ± 1.67 kg; Midwest Research Swine, Gibbon, MN) were housed for at least one week to allow for acclimation and testing of any preexisting disease. Prior to surgery, animals were fasted for 12 to 18 hours with access to water ad libitum. On the day of bone marrow aspirate (BMA), anesthesia was induced via 1–5% isoflurane (in 100% oxygen) and the pigs were endotracheally intubated. Catheters were placed in the left carotid artery and in the pulmonary artery (via left jugular vein), after which the pigs were maintained under total intravenous anesthesia (TIVA), comprised of ketamine HCl (20–30 mg/kg/hr) and midazolam HCL (1.0–1.5 mg/kg/hr) and propofol (100 mcg/kg/min) as described previously [36]. TIVA was carried out continuously and titrated to ensure full anesthesia and analgesia.

### 2.2. Bone Marrow Aspiration and Concentration

Five donor pigs were used for all assays (*n* = 5). Up to ten bone marrow samples were obtained from the tibia, femur, humerus, and iliac crest (i.e., bilateral) and five bone marrow samples from the sternum, for a total of nine bone marrow sites per animal. Prior to the aspiration, all materials were precoated with heparin to prevent clotting during collection and downstream processing. The area overlying each bone marrow site was aseptically prepared. A BMA needle (Arteriocyte Medical Systems, Hopkinton, MA) connected to a standard drill was inserted into the bone marrow compartment. The obturator was removed and a syringe, containing 8 ml of anticoagulant dextrose (ACD-A) solution (Arteriocyte Medical Systems), was attached and withdrawn to establish negative pressure. Aspirates were filtered to remove residual fat, bone chips, and clots. Approximately, 1 ml of BMA was set aside for cellular analyses and MSC characterization prior to concentration while the remainder was concentrated using the Magellan MAR01 System (Arteriocyte Medical Systems, Hopkinton, MA) according to the manufacturer's instructions. In instances where the volume of BMA was less than 30 ml, peripheral blood was added to the marrow component to accommodate the Magellan's minimal volume requirement. Following concentration, a similar volume of concentrated bone marrow aspirate (cBMA) was also used for comparative analyses.

### 2.3. Cellular Analyses and Mesenchymal Stem Cell Characterization

The volume of the BMA as well as concentration of nucleated cells and platelet retrieved from each bone marrow site was recorded. Nucleated cells and platelet numbers were obtained using the ADVIA® 120 Hematology System (Siemens, Malvern, PA). A subset of cells from BMA and cBMA was seeded onto plastic culture dishes in Dulbecco's Minimum Essential Media (DMEM) supplemented with 10% fetal bovine serum (Sigma-Aldrich, Saint Louis, MO), 2 mM L-glutamine, and 1% penicillin/streptomycin in a humidified incubator (5% CO_2_, 37°C). At subconfluency, cells were enzymatically detached with 0.25% trypsin–EDTA and characterized at passage 1 using the following assays: colony-forming unit fibroblast (CFU-F), multidifferentiation capacity, and flow cytometry.

#### 2.3.1. Flow Cytometry

Cultured cells were detached using Versene solution (Life Technologies, Carlsbad, CA) and collected in PBS. Samples were centrifuged at 1500 RPM for 10 min, washed with PBS, and centrifuged again. Cells were resuspended in BD Pharmingen Stain Buffer containing 2% fetal bovine serum (BD Biosciences, Franklin Lakes, NJ) and single cell suspensions were obtained by filtering cells through a cell strainer (40 micron pore size). As a control for positive CD45 expression, peripheral blood mononuclear cells (PBMCs) were isolated from whole blood using Ficoll-Paque Plus (GE Healthcare, Piscataway, NJ) per manufacturer's instructions. Viable cells were enumerated by trypan-blue exclusion using light microscopy. In a round-bottom 96-well plate, 2.5–5 × 10^5^ cells were added to each well. Purified mouse IgG antibody (SouthernBiotech, Birmingham, AL) was used to block cells at a concentration of 10 *μ*g per 10^6^ cells for 30 min at 4°C, and blocked cells were washed twice with Stain Buffer. Cells were incubated for 30 min at 4°C in 50 *μ*l of Stain Buffer containing an antibody cocktail. This consisted of CD90-APC (clone 5E10, Abcam, Cambridge, MA), CD105-PE (clone MEM-229, Abcam), and CD45-FITC (clone K252.1E4, AbD Serotec, Raleigh, NC); or isotype antibody cocktail containing mouse IgG1-FITC, IgG2a-PE, and IgG1-APC per manufacturer's recommendations. All isotype-control antibodies were purchased from BD Biosciences. Cells were washed twice with PBS and stained with BD Horizon Fixable Viability Stain 450 (BD Biosciences) per manufacturer's instructions. Cells were fixed in 1% paraformaldehyde, and data were acquired for 50,000 cells per sample using a MACSQuant flow cytometer (Miltenyi Biotec, San Diego, CA). Data were analyzed using FlowJo software (Tree Star, Inc., Ashland, OR).

#### 2.3.2. Colony-Forming Unit Fibroblast Assay

The colony-forming unit fibroblast (CFU-F) assay was used as an indicator of progenitor cell content in BMA and cBMA, as described previously [37]. Briefly, prior to the addition of peripheral blood (6.9 ± 1.7 ml) to the BMAs to accommodate the Magellan's minimal volume requirements, red blood cells were lysed with 2% acetic acid from each aliquot of BMA and nucleated cells counted manually using a hemocytometer. A total of 4 × 10^5^ nucleated cells were plated per well of a six-well plate in triplicates, and 3 ml of expansion media (DMEM supplemented with 10% fetal bovine serum, 2 mM L-glutamine, and 1% penicillin/streptomycin) was added. The media was changed every other day thereafter for 10 days. Cells were then washed with PBS and fixed with a 1 : 1 mixture of acetone : methanol for 10 min at room temperature. The plates were allowed to air dry; stained with Giemsa to allow for visualization; and colonies enumerated and reported as CFUs/ml. To gain an insight on MSC frequency in the MNC fraction obtained from the different sites, percent MSCs was calculated by dividing total CFUs by the total number of MNCs. Percent recovery of MSCs by the Magellan was calculated by dividing total CFUs in the cBMA by total CFUs in the BMA specimen and multiplying by 100.

#### 2.3.3. Multidifferentiation Assay

Adipogenic differentiation of cells was accomplished by replacing expansion media with preinduction adipogenic media consisting of DMEM, 10% FBS, 1% antibiotics, 0.5 mM isobutyl-methylxanthine (IBMX), 200 *μ*M indomethacin, 0.1 *μ*M dexamethasone, and 1 *μ*M insulin (Sigma-Aldrich, St. Louis, MO) for 24 hours, followed by two weeks of culture in adipogenic media (same as preinduction media minus the IBMX). Cells were fixed with 4% paraformaldehyde for 20 min and followed by staining with Oil Red O for one hour at room temperature (RT). Excess stain was removed by extensive washes with phosphate-buffered saline (PBS), and cells were imaged with an Olympus IX 71 inverted microscope.

Osteogenic differentiation of cells was achieved by replacing growth media with osteogenic media composed of DMEM, 10% FBS, 1% antibiotics, 10 mM *β*-glycerophosphate, 10 nM dexamethasone, and 150 *μ*M ascorbic acid 2-phosphate (Sigma-Aldrich, St. Louis, MO) for three weeks. Cells were fixed with 4% paraformaldehyde for 20 min, followed by staining with Alizarin Red S (40 mM, pH 4.1) for 20 min, and followed by extensive washes with water to examine mineralization activity. Images were collected as described above.

### 2.4. Statistical Analysis

Results are presented as means ± standard errors of the mean. All statistical tests were performed with the aid of GraphPad Prism version 5.00 and JMP version 10.0. A two-tailed Student's *t*-test was performed for a 2-group comparison. For more than two groups, a one-way analysis of variance (ANOVA) with a Tukey's Multiple Comparisons posttest was used to compare volume yield among the different bone marrow sites. In cases of unequal variances, a nonparametric, Wilcoxon test was performed. A *p* value of <0.05 was considered statistically significant.

## 3. Results

### 3.1. Cellular Analyses and Mesenchymal Stem Cell Characterization

**Figure 1:**
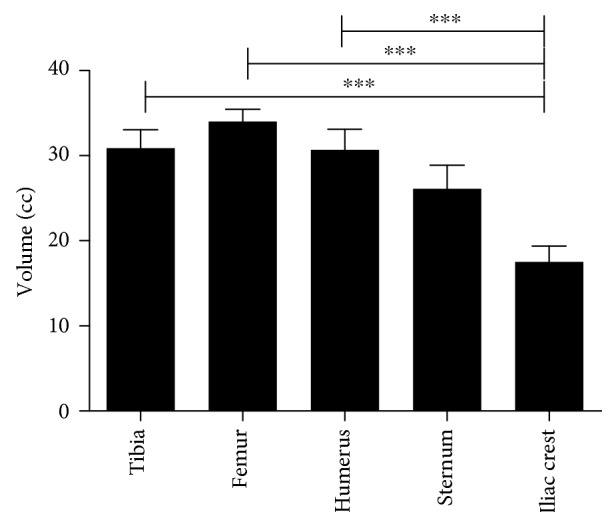
Volume yield of BMA from different bone marrow sites (*n* = 5); ^∗∗∗^*p* < 0.001.

**Figure 2:**
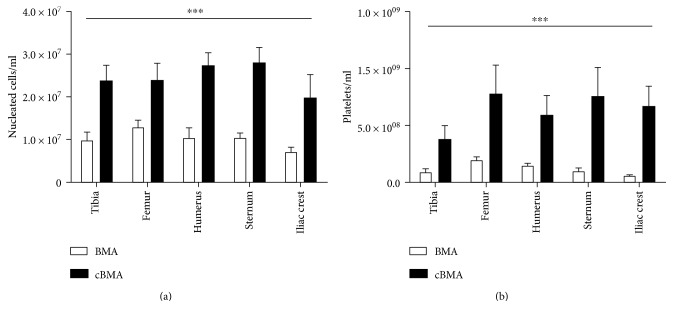
Concentration of nucleated cells (a) and platelets (b) obtained from different bone marrow sites. Significant differences were observed before (BMA) and after (cBMA) concentration, but not among the different sites; ^∗∗∗^*p* < 0.001.

The mean volume of BMA from each site is displayed in Figure 1. In terms of volume yield, significantly less bone marrow was retrieved from the iliac crests as compared to the other bone marrow sites (*p* < 0.001). Therefore, to accommodate the Magellan's minimal volume requirements, peripheral blood was added to the BMA prior to concentration at a mean volume of 14.7 ± 1.3 ml for the iliac crests and 5 ± 1.8 ml for the remaining bone marrow sites. The number of platelets and nucleated cells was also evaluated, and no significant differences were noted in their numbers among the different bone marrow sites. However, as expected, the concentration of nucleated cells and platelets increased significantly (*p* < 0.001) following processing with the Magellan (Figure 2).

**Figure 3:**
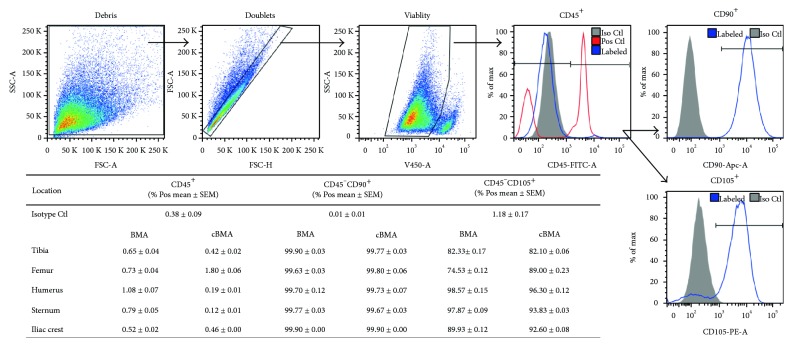
Mesenchymal stem cell surface markers (CD45^−^, CD90^+^, and CD105^+^) of cells isolated from multiple bone marrow sites before and after concentration.

Isolated cells from BMA and cBMA were also cultured and analyzed by flow cytometry for surface expression of the hematopoietic marker CD45 and MSC markers CD90 and CD105. The mean percentage ± SEM of cells that were CD45^+^, CD45^−^CD90^+^, and CD45^−^CD105^+^ were determined for each location of harvest (Figure 3). The majority of cultured cells isolated from all locations lacked the expression of CD45 as the average percentage of CD45^+^ cells was 0.75 ± 0.09 and 0.60 ± 0.31, pre- (BMA) and post-concentration (cBMA), respectively. The percentages of the MSC surface markers CD45^−^/CD90^+^ were 99.78 ± 0.05 for BMA and 99.77 ± 0.04 for cBMA, while CD45^−^/CD105^+^ were 88.65 ± 4.62 for BMA and 90.77 ± 2.47 for cBMA.

**Figure 4:**
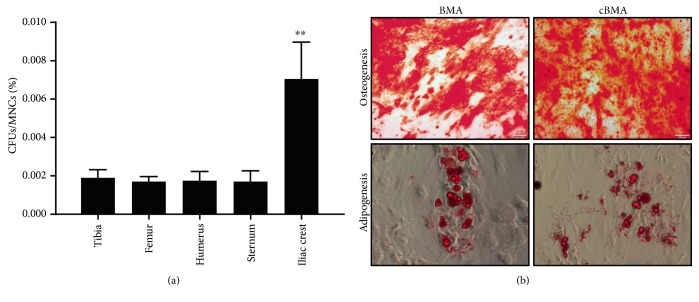
Percent MSCs among the different bone marrow sites prior to concentration (a) and multidifferentiation assays illustrating MSC characteristics of isolated cells from multiple bone marrow sites before and after concentration (b); ^∗∗^*p* < 0.01.

The CFU-F assay was used as an indicator of MSC presence in BMA and cBMA specimens. Similar to the nucleated cells and platelets, the number of CFUs significantly increased after concentration by the Magellan (*p* = 0.007). Overall, percent recovery of MSCs from the various sites via the Magellan was 51% ± 5.3 (mean ± SEM).  Table 1 presents CFU/well, CFU/ml, and total CFUs among the various bone marrow sites; interestingly, percent MSCs, as a fraction of total CFUs per MNCs prior to concentration, was significantly higher in the iliac crest (0.007%) compared to the other bone marrow sites (approximately 0.002%, *p* < 0.01, Figure 4(a)). Therefore, although the volume yield from the bone marrow of the iliac crest was significantly lower than other sites, it contained the highest concentration of MSCs.

To confirm the multilineage potential of cells isolated from the different bone marrow sites, culture-expanded cells derived from BMA and cBMA were induced via adipogenic and osteogenic media. After 21 days in osteogenic induction media, both BMA- and cBMA-derived MSCs showed nodule formation that stained positive for mineralization with Alizarin Red. Similarly, adipogenic differentiation was demonstrated by Oil Red O staining in BMA- and cBMA-derived stem cells after two weeks of induction (Figure 4(b)). No site variation was observed in the multidifferentiation capacity of the culture-expanded MSCs.

## 4. Discussion

The MSC is the most utilized stem cell type in clinical trials, due to its safety, ease of isolation, and robust regenerative capacity [5]. Although bone marrow harvesting from the iliac crest has long been the “gold standard” in humans due to the prominence of the iliac crest and ease of collection, only limited numbers of studies have compared MSC yield from different bone marrow sites. Hyer et al. compared the yield of MSCs obtained from BMA of the iliac crest, tibia, and calcaneus in humans. Consistent with our findings, they concluded that although all tested bone marrow sites contained progenitor cells, the iliac crest provided the greatest yield of MSCs [38]. McLain et al. reported similar or higher concentrations of MSCs from the vertebra as compared to the iliac crest when the two sites where compared in humans [39]. Henrich et al. compared the osteogenic function of MSCs recovered from BMA of the femur and iliac crest in humans. Their study demonstrated that MSCs obtained from the femur (using the reamer/irrigator/aspirator technique) exhibited significantly enhanced calcium deposition compared to the iliac crest [40]. As in the aforementioned studies, they only assessed the osteogenic differentiation capacity of the MSCs. Marx et al. evaluated the yield of CD34^+^, CD105^+^, and CD44^+^ cells from the tibial plateau, anterior, and posterior iliac crests in humans. They reported that both the anterior and posterior iliac crests had twice the amount of progenitor cells as the tibial plateau [41]. Beitzel et al. reported satisfactory concentrations of MSCs from proximal humerus and distal femur in humans [42]. Narbona-Carceles et al. then expanded on this work and compared MSCs isolated from the proximal tibia, distal femur, and iliac crest. They demonstrated that MSCs obtained from the three bone marrow sites possessed common MSC surface markers with multilineage differentiation capacity [43]. The published data presented above complements our findings, indicating that all bone marrow sites contain progenitor cells with multipotent characteristics, though the iliac crest possesses the highest frequency of MSCs.

Other than collection site, the quality of the bone marrow specimen and subsequent MSC yield can be affected from various factors, such as donor gender and age, BMA volume, rate and method of harvest, cell processing technique, and downstream cell-culture methods (Table 2). The *age of the donor* is fundamental, as aging has been correlated with depletion of the available stem cell pool [44–46]. In our study, the swine were not sexually mature (4–6 months old) and, thus, most likely possessed a large pool of stem cells, as demonstrated herein. The *aspirated volume* of the bone marrow sample has also been intimately linked with the frequency of isolated MSCs. Specifically, low-volume (i.e., 1–4 ml) BMA has been shown to contain higher concentrations of MSCs than high-volume aspirates due to increased dilution of the bone marrow specimen with peripheral blood [47, 48]. In our efforts to supplement this study and to optimize the approach for maximal MSC recovery, we have also compared 4 ml to 30 ml BMAs from the same iliac crests (bilateral). The 4 ml aspirates were processed using Ficoll-Paque while the 30 ml BMAs were processed using the Magellan. With respect to CFU-F assays and flow cytometry (CD45^−^, CD90^+^, CD105^+^, and CD73^+^), our preliminary findings indicate significantly higher concentration of MSCs in the smaller volume (4 ml) BMA samples (data not shown), similar to published literature. The *rate and magnitude of the applied negative pressure* also affects the quality of the BMA specimen. Herniguo et al. demonstrated that increased negative pressure coupled with low-volume aspirate and multiple insertion sites (in human iliac crest) resulted in higher concentrations of MSCs [47]. A similar conclusion was reached by Gronkjaer et al. showing that rapid aspiration, albeit more painful, is favorable over slow BMA for obtaining a high-quality bone marrow specimen [49]. In this study, the BMAs were aspirated into small volume syringes under high negative pressure.

Regardless of the optimal aspiration and postprocessing approach, variability in BMA quality and in MSC characteristics exists between donors [50, 51]. Phinney et al. showed that BMAs obtained from the iliac crests of 17 donors were highly variable in growth properties and in the osteogenic capacity of isolated MSCs, irrespective of gender and age [51]. Based on these published studies and our preclinical experience, we have learned that performing BMA procedures with 10 ml syringes minimizes variability and maximizes negative pressure for optimal MSC yield. Finally, the effect of the *cell processing method* will also have significant implications on the fate of isolated MSCs. As mentioned previously, the most common method for isolating MSCs is via density-gradient centrifugation, which has been demonstrated to recover a fraction of the MSC population [11–14]. We have found that percent recovery of MSCs using the Magellan was in the range of 36%–66% (mean 51% ± 5.3), which is a dramatic improvement over the Ficoll-Paque technique and constitutes an important outcome of our work. In our efforts to optimize MSC recovery, we further compared the Magellan to both RBC-lysed and direct plating of BMA to isolate MSCs. Data from our lab show that these approaches, and in particular direct plating of the BMA, are indeed more advantageous than other methods requiring downstream cell processing of the BMA sample. It is important to note, however, that irrespective of the bone marrow site or technique used, differences in MSC concentrations in the BMA sample are particularly pertinent when the primary objective is immediate administration to the patient (i.e., autologous treatment). This is because any apparent site/technique-related differences in the heterogeneity of the BMA sample will diminish with progressive cell culture, as the MSC population becomes more uniform [52]. Implications (if any) for long-term therapeutic potency of the stem cells may need to be further studied in light of the fashion in which they were retrieved and processed. However, based on the increased yield of MSCs after Magellan processing, we consider this device suitable as a bedside concentration tool for interventions requiring rapid administration of autologous bone marrow cells.

In this study, the BMAs were processed using the Magellan device, which unlike Ficoll-Paque rapidly generates a leucocyte-rich BMC sample. The BMC generated by the Magellan contains a variety of myeloid and lymphoid cells at various stages of differentiation. Nucleated cells and particularly platelets, in the form of platelet lysate or platelet-rich plasma, have been shown to potentiate the therapeutic milieu of the BMC [27, 28, 53]. Furthermore, the extended array of cytokines and growth factors most likely work in concert with the various bone marrow cells to facilitate the overall regenerative process in vivo [21, 28, 54–56]. In this study, the concentration of nucleated cells and platelets increased significantly (2.6-fold and 11-fold, respectively) following processing with the Magellan, a fact that may prove beneficial for autologous cell therapy. Following processing, culture-expanded cells from both BMA and cBMA expressed high levels of the MSC-surface markers CD90 (99.8%) and CD105 (89.8%) and demonstrated multilineage potential among all tested sites.

The Magellan system has also been evaluated in other studies for its ability to concentrate BMA. Rodriguez-Collazo et al. used the Magellan to concentrate BMA obtained from the proximal tibia in humans to expedite fracture healing. While they reported expedited fracture healing, the cellular products before or after processing were not evaluated [57]. Zhong et al. also used the Magellan to obtain BMC from humans and reported similar (2.8-) fold concentration for nucleated cells, as reported herein (2.6-fold). Analysis of hematopoietic and mesenchymal stem cell markers using flow cytometry (CD34^+^, CD271^+^, CD90^+^, CD105^+^, and CD146^+^) did not show an increase in stem cell concentration following processing [58]. Hegde et al. compared the Magellan to two other FDA-approved bone marrow concentration systems, namely Harvest SmartPrep 2 (Harvest Technologies Corporation, Plymouth, MA) and Biomet BioCUE (Biomet Biologics, Warsaw, IN), for their capacity to isolate progenitor cells from BMA of iliac crest in humans. The SmartPrep 2 system resulted in a greater concentration of progenitor cells, evaluated by means of a CFU-F assay, when compared to the Biomet and the Magellan system [25]. Cassano et al. performed extensive comparison on the composition of human BMC and platelet-rich plasma using also the Magellan and the SmartPrep 2. Unlike Hedge et al., they reported similar MSC concentrations in resultant BMC samples from the two systems, although the SmartPrep 2 yielded a higher concentration of nucleated cells [28]. They reported some instances of failure of the Magellan to concentrate the sample. We too have observed this phenomenon. We suspect that technical errors and variability of the obtained samples and their dilution with blood could all be responsible for concentration errors reported by others and us. Nonetheless, the improved MSC concentration potential of the Magellan suggests that it is suitable for point-of-care therapeutic applications when hematocrit, volume status, and collection conditions are well managed.

### 4.1. Limitations

The bone marrow specimens were harvested from swine, and thus extrapolation to humans should be done with caution. Although porcine MSCs are highly comparable to human MSCs in terms of their phenotype (such as for the positive expression of the common MSC surface markers CD90, CD105, CD73, CD29, CD44, etc.) [59, 60], and pigs share similar anatomic and physiologic characteristics with humans [61], interspecies variations in bone marrow and in blood rheology should be taken into consideration [62]. For example, the fact that the pig is a quadruped animal means different loads across its skeleton, these dissimilar skeletal loads may affect MSC fate through mechanisms such as mechano-transduction [63–65]. Another caveat is that the BMC samples generated with the Magellan are rich in platelets and leucocytes, the clinical implications of which have yet to be fully elucidated.

### 4.2. Conclusion

The rationale of this study was to identify the bone marrow site that is richest in MSC content. To this end, our results indicate that although MSCs were present in all tested bone marrow sites, the iliac crest, though low in bone marrow volume, possessed the highest frequency of MSCs. This fact is not only important for MSC isolation but also pertinent for autologous-based therapy. The second aim of this study was to evaluate the Magellan as a bone marrow concentration device for MSC collection. Compared to other isolation techniques, such as Ficoll-Paque, the cBMA generated by the Magellan contained a higher yield of MSCs suggesting that the Magellan has important potential advantages when rapid bedside administration of autologous BMC is the goal.

## Figures and Tables

**Table 1:** Colony-forming units (CFUs) among the different bone marrow sites before (BMA) and after (cBMA) concentration with the Magellan.

Bone marrow site (±SEM)	CFUs/well		CFUs/ml		Total CFUs	
	BMA	cBMA	BMA	cBMA	BMA	cBMA
Tibia	7.4 (±2.0)	22.2 (±6.3)	205.7 (±62.3)	1737.7 (±765.5)	9433.9 (±4065.1)	5213.1 (±2296.5)
Femur	7.0 (±1.3)	18.8 (±4.2)	206.3 (±85.6)	1593.6 (±555.2)	11235.2 (±3451.6)	4780.8 (±1665.5)
Humerus	6.5 (±2.3)	12.1 (±4.8)	210.4 (±76.8)	1129.3 (±468.9)	9347.3 (±3366.3)	3387.9 (±1406.6)
Sternum	7.2 (±2.2)	12.7 (±4.5)	203.5 (±91.7)	1404.0 (±641.6)	6338.8 (±1988.1)	4212.0 (±1924.8)
Iliac crest	25.6 (±11.2)	28.8 (±12.0)	623.7 (±282.5)	2295.6 (±849.1)	12692.3 (±4981.4)	6886.7 (±2547.2)

**Table 2:** Optimal conditions to maximize MSC yield from bone marrow aspirates (BMAs).

Factors	Optimal conditions	Reference
Gender & age	Young females	[44–46]
Bone marrow site	Iliac crest	[38–43]
BMA technique	Rapid with high negative pressure	[40, 47, 49]
BMA volume	Low volume	[47, 48]
Cell processing method	Direct bone marrow plating	[11–18]

## Abbreviations

BMA: Bone marrow aspirate
BMC: Bone marrow concentrate
cBMA: Concentrated bone marrow aspirate
CFU-F: Colony-forming unit fibroblast
DMEM: Dulbecco's Modified Eagle Medium
MSCs: Mesenchymal stem cells
PBMCs: Peripheral blood mononuclear cells
PBS: Phosphate-buffered saline.

## Additional Points

*Data Access.* The datasets during and/or analyzed during the current study are available from the corresponding author on reasonable request.
